# List of Recommended Names for bacteria of medical importance: report of the Ad Hoc Committee on Mitigating Changes in Prokaryotic Nomenclature

**DOI:** 10.1099/ijsem.0.006943

**Published:** 2025-10-23

**Authors:** Markus Göker, Henrik Christensen, Volker Fingerle, Marko Kostovski, Gabriele Margos, Edward R. B. Moore, Aharon Oren, Sheila Patrick, Udo Reischl, José A. Vázquez-Boland

**Affiliations:** 1 Leibniz Institute DSMZ – German Collection of Microorganisms and Cell Cultures, Inhoffenstrasse 7B, D-38124 Braunschweig, Germany; 2 Department of Veterinary and Animal Sciences, University of Copenhagen, Stigbøjlen 4, 1870 Frederiksberg C, Denmark; 3 Bavarian Health and Food Safety Authority, German National Reference Centre for Borrelia, Veterinärstr. 2, 85764 Oberschleissheim, Germany; 4 Institute of Microbiology and Parasitology, Faculty of Medicine, University Ss Cyril and Methodius in Skopje, Skopje, Macedonia; 5 Department of Infectious Disease, and Culture Collection University of Gothenburg, Institute for Biomedicine, Sahlgrenska Academy, University of Gothenburg, P.O. Box 7193, SE0492 34, Gothenburg, Sweden; 6 The Institute of Life Sciences, The Hebrew University of Jerusalem, The Edmond J. Safra Campus, 9190401 Jerusalem, Israel; 7 Wellcome-Wolfston Institute for Experimental Medicine, School of Medicine, Dentistry and Biomedical Sciences, Queen’s University Belfast, BT9 7BL Belfast, UK; 8 Institute of Clinical Microbiology and Hygiene, University Hospital Regensburg (UKR), Franz-Josef-Strauß-Allee 11, D-93053 Regensburg, Germany; 9 Microbial Pathogenomics Laboratory, Edinburgh Medical School and Institute for Regeneration & Repair, College of Medicine and Veterinary Medicine, University of Edinburgh, Edinburgh EH16 4UU, UK

**Keywords:** archaea, bacteria, databases, microbiology, standards, taxonomy

## Abstract

The Ad Hoc Committee on Mitigating Changes in Prokaryotic Nomenclature was recently established under the auspices of the International Committee on Systematics of Prokaryotes to address the impact of name changes of prokaryotic taxa in databases, scientific publications and other sources, particularly agencies responsible for establishing protocols and standards for infectious disease control. Here, we report on the activities of the committee and the actions taken to date. A first key task of the Committee was to emphasize to stakeholders that the vast majority of name changes are not mandatory under the International Code of Nomenclature of Prokaryotes (ICNP), and to provide guidance. The second key task was to compile a List of Recommended Names (LoRN) for bacteria of human or veterinary medical importance. This list has been incorporated into the List of Prokaryotic names with Standing in Nomenclature (LPSN) and is easily and freely available from LPSN. The principles for compiling and updating the list are described. Use of this list, rather than automatically treating the most recently proposed name of a taxon that has obtained standing under the ICNP as the name to be applied to the taxon, will greatly reduce the burden on practitioners who need to use prokaryotic names, e.g. in human and veterinary medical routines, and the risk associated with new, unfamiliar names. Other databases are encouraged to use LoRN.

## Introduction

Names used for the same taxon of prokaryotes may change in databases, journal articles and other sources, usually as a result of taxonomic activity proposing reclassifications [1,9]. Changing the name of a taxon of practical relevance can cause problems that can be broadly classified as ‘subjective’. This includes individual expectations associated with certain names but not with others, particularly unfamiliar ones; the need for practitioners to learn new, additional names and synonymy relationships and to adapt laboratory standard operating procedures and laboratory information systems; and the need for educators to adapt their curriculum to take account of such additional names and synonymy relationships. While it is certainly possible to overcome such subjective issues, it does take time and effort to adapt. However, changing a name can also cause ‘objective’ problems because of formal consequences, such as legal requirements or regulations, which may be linked to one (former) name for a taxon but not to another (more recent) name for the same taxon. For example, countries classify prokaryotes into risk groups [10] that determine which laboratories may handle which microorganisms. In addition, when the names of prokaryotes change, guidelines such as those issued by the Clinical and Laboratory Standards Institute, regulatory requirements for diagnostics and therapeutics and industry standards, such as pharmacopoeias, must be adapted. Changing the names of prokaryotes appears to be costly in several respects [11,16], raising questions of how important, urgent or mandatory name changes are in individual cases.

To address these issues, several preliminary meetings were held in 2023, including the Microbiology Society UK focused meeting in Glasgow in September 2023 [17], where a proposal to form a committee to address the issue of excessive name changes was presented in a talk. These meetings led to the formation of the Ad Hoc Committee on Mitigating Changes in Prokaryotic Nomenclature (CoMiCProN), which operates under the auspices of the International Committee on Systematics of Prokaryotes (ICSP) [9,18]. Members of CoMiCProN are human and veterinary clinical microbiologists, database managers and experts on systematics and taxonomic nomenclature. The first formal meeting of CoMiCProN was held on 30 January 2024. Initially, full meetings were held every 2 months, accompanied by optional bimonthly interim meetings; later in 2024, this was changed to one full meeting per month. The minutes of these meetings are available on the Zenodo platform [19], which makes them citable with a digital object identifier.

As a subcommittee of the ICSP, CoMiCProN conducts its activities in compliance with the International Code of Nomenclature of Prokaryotes (ICNP) [6], and some CoMiCProN members currently hold other positions within the ICSP. The starting point for CoMiCProN was therefore to explore the possibilities for mitigating name changes provided by the official rules of prokaryotic nomenclature. Early on, it was emphasized that the ICNP does not make name changes mandatory in most circumstances, particularly in those common cases wherein a name change reflects the placement of a species in a different genus than before [1,5,7]. Indeed, this is a widespread misunderstanding of the remit of the ICNP, perpetuated by the practices of some databases [20]. CoMiCProN therefore concluded as an important line of action that selected scientific journals should be contacted and informed about such relevant misunderstandings in prokaryotic nomenclature. This activity was included in the CoMiCProN mission statement, and the letters to journals formed the basis for the CoMiCProN guidelines published online [21].

Of course, it may help to avoid unwarranted name changes if one knows that the name of a taxon that has most recently received standing in the ICNP [6] may or may not be considered the name to be used for that taxon. However, this realization immediately leads to the question of which name to choose. Obviously, a centralized resource for names to be applied to a taxon could save a lot of time and effort compared to, for example, individual naming decisions by each practitioner, institution or database manager working with prokaryotic taxa, and also facilitate communication about these taxa. This is despite the fact that such a centralized resource can only provide recommendations on taxonomy according to the ICNP [6], which supports ‘freedom of taxonomic thought or action’. CoMiCProN has therefore included in its mission statement the goal of providing recommendations for the naming of prokaryotes of practical relevance, with the human and veterinary medical fields being of primary interest.

Compiling a List of Recommended Names (LoRN) for bacteria of human or veterinary medical importance requires a workflow that takes, as input, a set of synonymous names that have standing under the ICNP [6] and selects the one to be recommended for practical applications. The workflow, in turn, requires a list of concepts that are generally applicable to all situations that may arise when a choice has to be made between alternative synonymous names.

The first list of such concepts (and terms for them) was announced to CoMiCProN on 30 January 2024, while the first draft of the complete workflow stemming from these concepts and terms was presented on 2 April 2024 [19]. This was followed by a discussion of examples of the application of the workflow, which, in turn, led to the formal approval in principle of the two-step approach of the workflow on 4 June 2024 and the ratification of the first and second steps of the approach on 6 August 2024 [19]. Since then, several drafts of LoRN have been discussed, and outstanding issues have been resolved, focusing on the treatment of names for individual taxa, as some implementation details are deliberately left open by the workflow to allow for consideration of information specific to the practical consequences of a particular taxon. This work resulted in the first comprehensive and integrated version of LoRN on 4 March 2025 [19] and the publication of the workflow on Zenodo on 2 May 2025 [22]; this file is included as File S1, available in the online Supplementary Material. However, a formal announcement of LoRN, together with a description of how best to use it and what the underlying principles of its workflow are, was not previously finalized.

This report addresses this gap and is structured as follows. Firstly, it explains how to obtain and use LoRN, as these are the issues of most practical relevance. This first section should provide an easy orientation for anyone, whether a practitioner interested in checking the names of individual bacteria or a database manager interested in mass importation of recommended names and their synonyms. Secondly, the principles underlying the creation of LoRN are outlined. This second section is a more in-depth exploration of the issues involved, necessarily presupposing knowledge of prokaryotic taxonomy and nomenclature and, therefore, may not be immediately accessible to everyone. However, its first subsection reiterates why the last validly published name among a set of synonyms is not necessarily the correct name, something that anyone concerned with prokaryotic nomenclature should be aware of. Thirdly, selected taxonomic consequences are drawn further to illustrate the application of the principles outlined above. Finally, the future of LoRN is outlined, particularly with regard to regular updates and user feedback.

## How to obtain and use LORN

In order to make LoRN easy to obtain and keep up-to-date, CoMiCProN has partnered with the List of Prokaryotic names with Standing in Nomenclature (LPSN) [5]. LPSN has been available since 1997 [23,25] to provide detailed and updated information on prokaryotic nomenclature in compliance with the ICNP [6]. Since 2020, LPSN has been hosted by the Leibniz Institute DSMZ – German Collection of Microorganisms and Cell Cultures [26], a state-funded non-profit organization. As of 2023, LPSN is recognized as a Global Core Biodata Resource [5]. To support CoMiCProN, LPSN has collected a large amount of additional data, including risk group classifications from several countries, evidence of human, animal or plant pathogenicity from the collected literature and information on whether strains assigned to one species have previously been assigned to another, non-synonymous species. This information is also displayed on the LPSN pages for individual taxon names and is accessible via the LPSN advanced search [5]. In addition, where necessary, LPSN has adapted its taxonomy to the CoMiCProN workflow described below.

An initiative for addressing nomenclatural issues of pathogenic fungi [27], whose aims and activities are similar to those of CoMiCProN, has been criticized for resulting in a new database of taxon names in addition to already established databases [28]. In contrast, by collaborating with LPSN, which is already widely used by researchers in various fields of microbiology, including clinical microbiologists, CoMiCProN has avoided the need for a separate database. CoMiCProN encourages other databases to make use of LoRN and hopes that the ease of access via LPSN will contribute to the adoption of the recommended names as the correct names of the taxa concerned. If other databases adopted the recommended names, their end users would not need to use LPSN for this purpose and would not even need to know about LoRN.

**Fig. 1.**
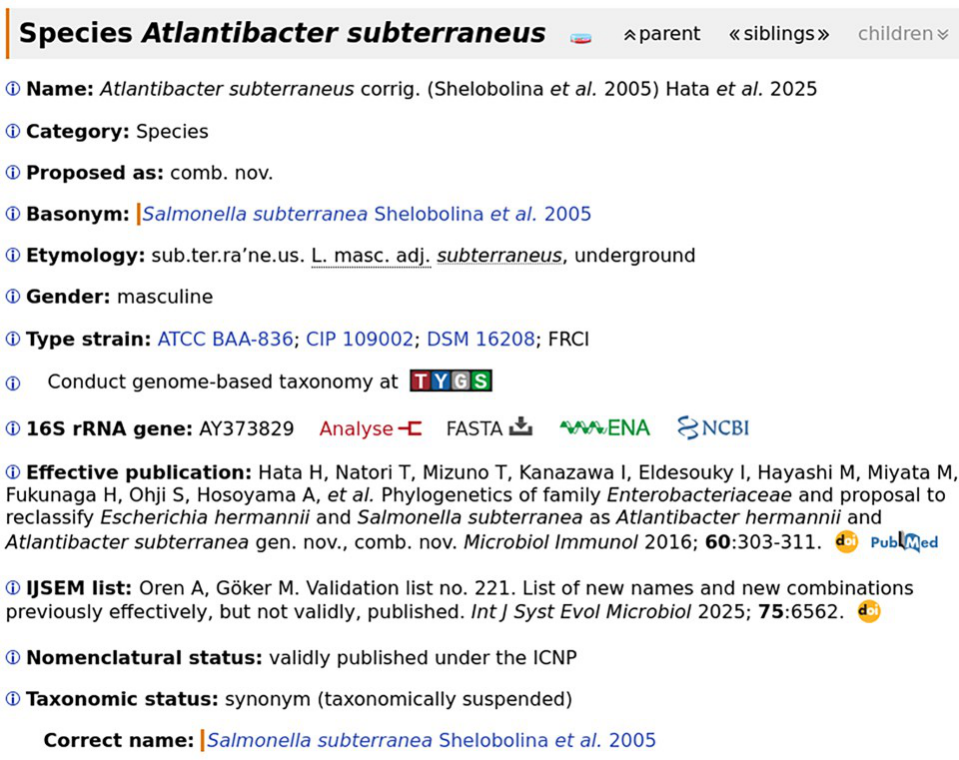
Screenshot of LPSN [5] taken on 8 May 2025. The LPSN page for this taxon name, *Atlantibacter subterraneus* corrig. (Shelobolina *et al.* 2005) Hata *et al.* 2025 [31,32], indicates that it is not a recommended name. This also implies that LPSN does not treat it as the correct name but as a synonym. In this particular case, it is not the correct name because it is in taxonomic suspension. This means that *Salmonella subterranea* Shelobolina *et al.* 2005 [29,30] should be used instead until the newer name has obtained the same salience. Please note that taxonomic suspension is listed under taxonomic status, whereas nomenclatural status is defined by the ICNP. Users can click on the ⓘ symbol for more information.

The main mechanism by which LPSN implements LoRN is straightforward (Fig. 1): LPSN treats the recommended name for a taxon as its correct name under the ICNP [6]. In other words, the set of names recommended by LoRN is the intersection of the set of correct names in LPSN and the set of names in LoRN. To ensure that they can be mapped to the correct names, synonyms are also included in LoRN. Bacterial names in LoRN are marked as such (Fig. 1) and can be found via the LPSN advanced search and via the LPSN Application Programming Interface. Moreover, LoRN-specific actions described below, such as placing a name in taxonomic suspension in order to maintain another one as the correct name, which is a temporary measure, are indicated as such in LPSN. In this way, LPSN makes LoRN accessible to everyone, and the creation of a LoRN-specific database can be avoided as long as LPSN cooperates with CoMiCProN.

## Principles for compiling LoRN

### The last validly published name need not be the correct name

It is not infrequent that users of prokaryotic names believe in what the workflow calls the taxonomic *argumentum ad novitatem* (appeal to novelty) [22], mistakenly assuming that the official rules actually state that the last validly published and legitimate name (among a set of homotypic synonyms) must be treated as the correct name [21]. However, the ICNP [6] makes no such statement and, in fact, advocates taxonomic freedom [1,5,7]. According to Rule 23a Note 5 of the ICNP [6], the correct name is ‘the name which must be adopted for a taxon under the Rules’. As stated in Principle 8, the correct name depends on circumscription and position:

‘Each phylum or taxon of a lower rank with a given circumscription, position, and rank can bear only one correct name … Circumscription of the taxon is an indication of its limits; … position of a taxon is an indication in which higher taxon it is placed.’

However, General Consideration 4 informs us that ‘Rules of nomenclature do not govern the delimitation of taxa nor determine their relations’, meaning that the ICNP does not regulate circumscription and position; they can therefore be chosen if several validly published and legitimate names are available [1,7, 8]. For the species shown in Fig. 1, a position in the genus *Salmonella* has been chosen to allow the name *Salmonella subterranea* Shelobolina *et al.* 2005 [29,30] to be treated as the correct name in place of *Atlantibacter subterraneus* corrig. (Shelobolina *et al.* 2005) Hata *et al.* 2025 [31,32].

Unfortunately, treating the last validly published name as the correct name is also the routine practice of some databases [20], probably because it is attractive to them in being easy to implement. This, in turn, may lead users to believe that there are official rules enforcing it.

In contrast, CoMiCProN [21,22] emphasizes that users of taxon names should be aware of the principle of the taxonomic freedom implemented in the ICNP [6]. This extends to journals, databases, agencies and service providers, which should be aware of the taxonomic freedom principle and should not report ‘name changes’ as such, except in the few cases enforced by ICNP [6,7]. This also helps to implement LoRN for prokaryotes that does not automatically treat the last validly published and legitimate name (among a set of homotypic synonyms) as the correct name but distinguishes between taxonomic reclassifications that are sufficiently important to be accepted and reclassifications that are not important enough to justify the resulting name changes (Table 1). Another feature allowed by taxonomic freedom is a ‘grace period’ before names are replaced due to acceptance of reclassifications. Applying these principles, changes in prokaryotic nomenclature can be mitigated effectively.

**Table 1.** Differences between taxonomic views expressed in the order of their decision criteria An antitaxonomic stance usually implies discarding empirical (e.g. phylogenetic) results in order to achieve absolute name stability. At the other extreme, hypertaxonomic views show the least concern for name stability. A taxonomically conservative approach lies in the middle.

Approach	Ordering of criteria
Antitaxonomic	Stability of names>monophyly>thresholdism/signaturism
Taxonomically conservative	Monophyly>stability of names>thresholdism/signaturism
Hypertaxonomic	Monophyly>thresholdism/signaturism>stability of names

### Not all taxonomic reclassifications are important enough to be adopted

The workflow only considers genus, species and subspecies ranks and the splitting, merging and otherwise rearranging of taxa at these ranks [22]. The workflow defines its own approach as parataxonomic (from Gr. prep. *para*, beside), thereby emphasizing that CoMiCProN does not intend to interfere in any way with the taxonomic activities of others [22]. This is fully in line with the ICNP, which does not regulate taxonomy and supports taxonomic freedom [6,7]. Instead, the first step in the workflow is to review the given taxonomic reclassification proposals and filter out those that are not considered important enough to warrant a name change. Those reclassifications that do not pass this filter are therefore not recommended by LoRN [22].

According to Article 3(a) of its Statutes [18], the ICSP must represent the diverse interests of the various microbiological disciplines in prokaryotic nomenclature. This requires, for example, taking into account the interests of practitioners as well as taxonomists. The first step of the parataxonomic approach implemented by the workflow is therefore intended to be a compromise, positioned fairly precisely between the possible extremes of views on taxonomy [22]. To prove this, these extremes need to be named and characterized. Of course, the extremes must also be taxonomic approaches or viewpoints, and they must actually occur in the scientific literature. The main question of CoMiCProN is how to mitigate name changes [19,21], whereas taxonomy, besides being a theoretical concept, is also an empirical science. For this reason, taxonomic views should be ranked according to the likelihood that the way in which they derive taxonomies from empirical data will lead to reclassifications, as these in turn may imply name changes (Table 1).

Absolute name stability can, of course, be achieved by ignoring empirical analyses that imply a need for reclassification. This viewpoint does exist, with a particularly instructive example being the Request for an Opinion [33] that was denied by the Judicial Commission of the ICSP in Judicial Opinion 122 [3]. The workflow calls this the antitaxonomic viewpoint [22]. Another manifestation of antitaxonomic thinking is the preference of a taxonomic approach that is virtually never used over much more common approaches, simply on the assumption that the former will avoid name changes in a small group of organisms of interest [34]. In general, one would expect it to be more persuasive to do one’s best to fit one’s conclusions to one’s premises, rather than to try to fit one’s premises to one’s conclusions. Furthermore, decades of literature on the subject agree that taxonomic classification should have a unifying goal [35,42], and we doubt that this comes as a surprise.

With respect to taxonomic approaches that do not reject empirical data, the question arises as to their common denominator. Clarifying this requires a historical assessment of different schools of taxonomy [35,42]. Among the three major schools of taxonomic thought, ‘phenetics’ groups by overall similarity rather than phylogeny, ‘evolutionary taxonomy’ is based on phylogenies and does not allow polyphyletic taxa but does allow some paraphyletic taxa and ‘phylogenetic systematics’ is based on phylogenies and only allows monophyletic taxa. Historically, phylogenetic systematics has prevailed over phenetics because the latter does not adequately reflect biological evolution. Phylogenetic systematics has also prevailed over evolutionary taxonomy because, if the unifying goal of taxonomic classification is to summarize phylogeny, it can be formally demonstrated that this implies the need for monophyletic taxa throughout [35,42]. Thus, the common denominator is monophyly.

Taxonomic conservatism is therefore defined by the workflow [22] as the approach that consistently aims for monophyletic taxa and repairs non-monophyletic taxa in a way that results in the lowest number of reclassifications and therefore the lowest number of name changes [42,45]. In the workflow, approaches that use criteria other than minimizing name changes – after monophyly as the primary criterion – are called hypertaxonomic (Table 1). Among these, signaturism is defined as the idea that the chosen monophyletic groups must also have particular character signatures, such as particular sets of ‘conserved signature indels’ [22]. In practice, signaturism has led to the splitting of genera that were already monophyletic even according to the studies proposing their split [46,47]. Such approaches have been criticized because sometimes a split genus was not only monophyletic but also showed character signatures [43]. Apart from that, any criterion other than name stability, if placed second after monophyly, will, on average, lead to more name changes than taxonomic conservatism, for obvious reasons (Table 1).

This also applies to thresholdism, defined as the idea that the selected monophyletic groups must have a certain degree of divergence, such as a maximum phylogenetic depth [22]. In practice, the application of thresholdism has led to numerous reclassifications, even within a single study [48] and even when based on a supposedly conservative approach [49]. It has already been noted that this approach is not taxonomically conservative, partly because the thresholds chosen have not been optimized to minimize the number of resulting name changes [42] and partly because an approach more conservative than thresholdism itself can easily be devised (Table 1). While thresholdism has the advantage of making taxa of the same rank quantitatively comparable, it cannot make taxon delimitation objective [42]. There are no true thresholds of, say, genera in nature that could be approximated by the taxonomist [42]. Of course, it can be observed that, e.g. families have, on average, a larger character divergence than genera. However, this is either a by-product of the Linnaean hierarchy used by codes such as the ICNP [6], wherein genera are placed in families but not vice versa, or the result of previous attempts to apply thresholdism, which would lead to a circular argument [42].

The existence of different approaches to derive taxonomies from empirical data also proves that a newer classification, even if it is based on more or better data, need not be different, for that reason, from an earlier classification of the same organisms. The belief that newer classifications are better because they are based on more or better data is one of the possible reasons for the taxonomic *argumentum ad novitatem* [22]. However, this belief is an example of another logical fallacy, the *post hoc ergo propter hoc* fallacy [50]. A study proposing a new classification may indeed be able to use more or better data than previous publications, but this does not mean that the new classification is implied by the greater amount or quality of the data. The study may also use a new taxonomic concept that does not follow logically from the data, such as thresholdism or signaturism [22]. In fact, what should be done (e.g. how to classify taxonomically) never follows only from what is [51,53].

For a committee that attempts to mitigate name changes (instead of fostering or abolishing them) [19], taxonomic (re-)classification must reflect empirical data in a unified way that minimizes name changes. Some name changes cannot be avoided, but the effect of these situations can also be mitigated. The parataxonomic approach therefore builds on taxonomic conservatism, as step 1, and adds, as step 2, a process of deferring the application of some taxonomically preferred names until some requirements of practical relevance are likely to be met [22], which is described below. At the species level, the parataxonomic approach makes use of thresholdism, because the 70% DNA:DNA hybridization criterion [54,56] has been used almost universally in microbiology [42,57, 58], even before the publication of the Approved Lists [59], which was the starting point for current prokaryotic nomenclature [6]. This does not mean that CoMiCProN considers this criterion or its modern equivalents [60,63] to be objective, but that the committee believes that retaining these criteria will lead to fewer name changes overall.

### Some name changes should be postponed

There are name changes that practitioners should eventually accept. These include changes from not validly published names to validly published names and from illegitimate names to legitimate names, which are required by the ICNP [6,8] but are relatively rare. As noted above, name changes resulting from taxonomic revisions are also acceptable if the revisions are taxonomically conservative [22]. As such, name changes have costs, i.e. multiple negative practical consequences at different levels. The second step of the workflow is to assess whether an acceptable name change should be deferred. The underlying concepts are called taxonomic suspension, salience and known practical implications [22].

The salience (or prominence) of a taxon name depends on how well it is represented in official documents that have practical implications. For example, if an older name is associated with legal requirements, regulations or restrictions [10], and its proposed replacement has not yet been evaluated by the appropriate regulatory agency, then the older name will have considerably greater salience. It may be that a reclassification was based on taxonomic conservatism and was therefore accepted in step 1 of the workflow [22], but the newer and older names differ in salience. Such names are therefore considered for taxonomic suspension (Fig. 1).

Different bacterial names resulting from a clinical diagnosis may lead to different treatments for the patient, such as the choice of antibiotics. This is an example of names with considerably different known practical implications. In such situations, the more recent name will not be placed in taxonomic suspension. It may also be the case that one of the names designates a pathogen and the other does not. For example, the recently proposed *Rhodococcus parequi* Vazquez-Boland *et al.* 2025 [64] contains strains that conventional identification tests would place in *Rhodococcus equi* (Magnusson 1923) Goodfellow and Alderson 1977 (Approved Lists 1980) [59,65]. However, unlike *R. equi*, *R. parequi* is not considered to be pathogenic [64]. Otherwise, *R. parequi* might have been placed in taxonomic suspension and temporarily treated as a later heterotypic synonym of *R. equi*.

If the names differ considerably in salience but do not differ considerably in known practical implications, however, the newer name will be placed in taxonomic suspension [22]. An example of a name in taxonomic suspension is *Bacteroides hominis* Liu *et al.* 2022 [66,67]. Strains assigned to this species were previously assigned to *Bacteroides fragilis* (Veillon and Zuber 1898) Castellani and Chalmers 1919 (Approved Lists 1980) [59,68], a much better-known name for a species for which no practical differences from *B. hominis* are currently known.

For the current versions of LoRN, a considerable difference in salience is assumed if the newer name has a lower risk group than the older name in the majority of risk group classifications imported into LPSN [5]. If the risk group is not indicated, the bacterium is assumed to be classified in Risk Group 1 (not associated with disease in healthy adults), resulting in a difference in salience being reported if the older name has a risk group classification >1. The names selected for taxonomic suspension are cases of potential or established pathogens where the newer names have a lower risk group or (mostly) no indicated risk group in most of the risk group classifications imported [10]. Taxa not considered by LPSN to be potential or established pathogens were not selected for taxonomic suspension. Newer names more than 5 years old were also not selected for taxonomic suspension. In these cases, it was assumed that the agencies compiling the risk group classifications simply had difficulty in keeping them up to date. Such implementation details are not fixed in the workflow [22] and can therefore, in principle, be changed after negotiation with users and other stakeholders. However, it is necessary to ensure that taxonomic suspension ends; otherwise, the approach would effectively be the same as the antitaxonomic approach (Table 1), which has been explicitly rejected and seems incompatible with Article 3(a) of the ICSP Statutes [18].

The workflow assures that names in taxonomic suspension will eventually be accepted and that users of LoRN will therefore be informed in advance of the impending changes [22]. Names that have been considered for taxonomic suspension but have not been taxonomically suspended are also highlighted, as this informs users of past name changes of practical relevance.

## Taxonomic consequences

It may seem odd that a committee designed to mitigate changes in nomenclature should itself attempt to cause such changes. However, name changes are mainly caused when databases and other agencies, or large numbers of individual users of taxon names, adopt reclassifications. The reversal of these changes may itself be supported by name changes. As indicated above, a committee that aims to mitigate changes in nomenclature should embrace taxonomic conservatism. As a result, the taxonomic classification at the genus level endorsed by the committee may differ from alternative classifications favoured in some publications. This could lead to the creation of what LPSN refers to as ‘orphaned species’ [5] when the committee’s genus-level classification is implemented. This, in turn, can be remedied by proposing new combinations that reflect the committee’s genus classification.

As mentioned above, CoMiCProN does not agree with the policy of some databases to always treat the last validly published name among a set of homotypic synonyms as the correct name. Although this policy is easy to implement, it can cause problems from a user perspective [20]. It should be added that such an approach can be problematic even on purely taxonomic grounds. In an era of competing taxonomic views on the circumscription of a genus, some of the last validly published names of species among a set of homotypic synonyms associated with that genus may reflect the taxonomic view that the genus should be retained, while others may reflect the view that the genus should be split into several genera. Treating the last validly published name among a set of homotypic synonyms always as the correct name may therefore lead to taxonomic confusion. The taxonomic work of a committee designed to mitigate changes in nomenclature should therefore seek to implement taxonomic conservatism [22] and reduce the potential for this type of confusion by highlighting that it is caused by a policy that might better be abandoned.

Currently, the proposal to split *Borrelia* Swellengrebel 1907 (Approved Lists 1980) [59,69] by assigning some species to *Borreliella* Adeolu and Gupta 2015 [46,70] and the proposal to split *Mycobacterium* Lehmann and Neumann 1896 (Approved Lists 1980) [59,71] into a total of five genera [47,72] are the most prominent examples of reclassifications of medically relevant genera that were not based on taxonomic conservatism [22]. Another example is the new genus *Prescottella* Sangal *et al.* 2022 [73,74], proposed for the sublineage of *Rhodococcus* Zopf 1891 (Approved Lists 1980) [59,75] containing the animal and human pathogen *R. equi* (Magnusson 1923) Goodfellow and Alderson 1977 (Approved Lists 1980) [59,65]. The nested genus *Prescottella* makes the genus *Rhodococcus* paraphyletic [76].

In the three cases, the genera proposed to be split were already monophyletic, even according to the phylogenetic analyses performed in the studies proposing the split [46,47]. While several articles have been published in favour of [77,78] or against [11,14, 79,82] splitting *Borrelia*, there are currently no known ‘orphaned species’ of *Borreliella*, as all *Borreliella* species names have validly published and legitimate basonyms in the genus *Borrelia* that may be used in their place [5] and are, of course, preferred in LoRN [22]. The same holds for *Rhodococcus*. In contrast, several ‘orphaned species’ names are validly published in *Mycolicibacter* Gupta *et al.* 2018 and *Mycolicibacterium* Gupta *et al.* 2018 [47,72] because they were proposed as new species rather than new combinations with *Mycobacterium* spp. basonyms [47,83,100]. The application of taxonomic conservatism [22] and the criticism raised against splitting *Mycobacterium* [13] call for proposing new combinations to assign these species to *Mycobacterium*.

The genus *Raoultella* Drancourt *et al.* 2001 [101,102] was proposed to include some species previously placed in *Klebsiella* Trevisan 1885 (Approved Lists 1980) [59,103], based on a 16S rRNA gene tree with low bootstrap support, a proposal not consistent with taxonomic conservatism. Ma *et al.* [104,105] showed that the genus *Raoultella* is nested within the paraphyletic genus *Klebsiella* and proposed to reclassify *Raoultella electrica* Kimura *et al.* 2014 [106,107] as *Klebsiella electrica* (Kimura *et al.* 2014) Ma *et al.* 2022 [104,108]. It follows that the species of *Raoultella*, including its type species, that were originally classified with *Klebsiella* may be referred to with their original *Klebsiella* species name as the correct name, as they are homotypic synonyms. Recently, however, Brady *et al.* [109] proposed the names of two new species, *Raoultella lignicola* Brady *et al.* 2024 and *Raoultella scottii* corrig. Brady *et al.* 2024. If *Raoultella* is considered to be a later heterotypic synonym of *Klebsiella*, as shown by Ma *et al.* [104,105], the new species *R. lignicola* and *R. scottii* become ‘orphaned species’. Since even the phylogenetic tree published by Brady *et al.* [109] showed that the *Raoultella* species are nested within *Klebsiella*, they are classified here as new combinations within *Klebsiella*.

## Description of *Klebsiella lignicola* (Brady *et al.* 2024) comb. nov.

lig.ni’co.la. L. neut. n. *lignum*, wood; L. masc./fem. n. suff.-­*cola*, dweller; N.L. fem. n. *lignicola*, wood inhabitant.

Basonym: *Raoultella lignicola* Brady *et al.* 2024 [109,110].

The description is given as for the basonym [109]. The type strain is TW_WC1a.1 (=CCUG 77094=LMG 33073).

## Description of *Klebsiella scottii* (Brady *et al.* 2024) comb. nov.

scot’ti.i. N.L. gen. masc. n. *scottii*, named after Lewis Scott for his contribution and support of Acute Oak Decline research.

Basonym: *Raoultella scottii* Brady *et al.* 2024 [109,110].

The description is given as for the basonym [109]. The type strain is BAC 10 a-01-01 (=CCUG 77096=LMG 33072).

## Description of *Mycobacterium acapulcense* (Gupta *et al.* 2024) comb. nov.

a.ca.pul.cen’se. N.L. neut. adj. *acapulcense*, from Acapulco, a town on the Pacific coast of México.

Basonym: *Mycolicibacterium acapulense* Gupta *et al.* 2024 [47,99].

The description is as given for the basonym [47]. The type strain is AC-103 (=ATCC 14473=CECT 3026=JCM 6402). We correct the spelling of the epithet from *acapulense* to *acapulcense*, as *acapulense* appears to be a typographical error, according to the etymology given by Gupta *et al.* [47].

## Description of *Mycobacterium acidiphilum* (Xia *et al.* 2022) comb. nov.

a.ci.di’phi.lum. N.L. neut. n. *acidum*, an acid; from L. masc. adj. *acidus*, sour; N.L. masc. adj. suff. *-philus*, friendly to, loving; from Gr. masc. adj. *philos*, friendly to, loving; N.L. neut. adj. *acidiphilum*, acid-loving.

Basonym: *Mycolicibacter acidiphilus* Xia *et al.* 2022 [91,93].

The description is as given for the basonym [93]. The type strain is M1 (=KCTC 49392=MCCC 1H00416).

## Description of *Mycobacterium arseniciresistens* (Zhu *et al.* 2024) comb. nov.

ar.se.ni.ci.re.sis’tens. L. neut. n. *arsenicum*, arsenic; L. pres. part. *resistens*, resisting; N.L. neut. part. adj. *arseniciresistens*, arsenic-resisting, referring to the arsenic resistance of the bacterium.

Basonym: *Mycolicibacterium arseniciresistens* Zhu *et al.* 2024 [97,100].

The description is as given for the basonym [97]. The type strain is KC 300 (=CGMCC 1.19494=JCM 35915).

## Description of *Mycobacterium aurantiacum* (Pan *et al.* 2022) comb. nov.

au.ran.ti’a.cum. N.L. neut. adj. *aurantiacum*, orange.

Basonym: *Mycolicibacterium aurantiacum* Pan *et al.* 2022 [83,92].

The description is as given for the basonym [92]. The type strain is B3033 (=KCTC 49712=MCCC 1K04526).

## Description of *Mycobacterium baixiangningiae* (Cheng *et al*. 2021) comb. nov.

bai.xiang.ning’i.ae. N.L. gen. fem. n. *baixiangningiae*, of Dr Xiangning Bai, for her great contribution to the study of isolation and identification work in novel species among Qinghai-Tibet Plateau samples.

Basonym: *Mycolicibacterium baixiangningiae* Cheng *et al.* 2021 [87,90].

The description is as given for the basonym [87]. The type strain is LJ126 (=CGMCC 1.1992=KCTC 49535).

## Description of *Mycobacterium cyprinidarum* (Matsumoto *et al.* 2024) comb. nov.

cy.pri.ni.da’rum. N.L. gen. fem. pl. n. *cyprinidarum*, of the *Cyprinidae*.

Basonym: *Mycolicibacterium cyprinidarum* Matsumoto *et al.* 2024 [96,98].

The description is as given for the basonym [96]. The type strain is NGTWS1803 (=ATCC TSD-289=JCM 35117).

## Description of *Mycobacterium lacusdiani* (Xiao *et al.* 2023) comb. nov.

la.cus.di.a’ni. L. masc. n. *lacus*, a lake; N.L. gen. masc. n. *lacusdiani*, of Dian lake.

Basonym: *Mycolicibacterium lacusdiani* Xiao *et al.* 2023 [94,95].

The description is as given for the basonym [94]. The type strain is JXJ CY 35 (=CGMCC 1.17501=KCTC 49379).

## Description of *Mycobacterium mengxiangliae* (Cheng *et al.* 2021) comb. nov.

meng.xiang.li’ae. N.L. gen. fem. n. *mengxiangliae*, of Dr Xiangli Meng, for her great contribution to the study of isolation and identification work in novel species among Qinghai-Tibet Plateau samples.

Basonym: *Mycolicibacterium mengxianglii* Cheng *et al.* 2021 [87,90].

The description is as given for the basonym [87]. The type strain is Z-34 (=CGMCC 1.1993=DSM 106172). We have corrected the epithet from *mengxianglii *to *mengxiangliae* because the former implies a male person according to Appendix 9 of the ICNP [6].

## Description of *Mycobacterium nivoides* (Dahl *et al.* 2021) comb. nov.

ni.vo.i’des. L. fem. n. *nix*, snow; L. adj. suff. *-oides*, resembling, similar; from Gr. neut. adj. suff. *-eides*, resembling, similar; from Gr. neut. n. *eîdos*, that which is seen, form, shape, figure; N.L. neut. adj. *nivoides*, snow-like.

Basonym: *Mycolicibacterium nivoides* Dahl *et al.* 2021 [84,89].

The description is as given for the basonym [84]. The type strain is DL90 (=JCM 32796=NCCB 100660).

## Description of *Mycobacterium stellerae* (Nouioui *et al.* 2019) comb. nov.

stel’le.rae. N.L. gen. fem. n. *stellerae*, of *Stellera*, named referring to the host plant, *Stellera chamaejasme*, from which the strain was isolated.

Basonym: *Mycolicibacterium stellerae* Nouioui *et al.* 2019 [85,86].

The description is as given for the basonym [85]. The type strain is I10A-01893 (=CECT 8783=DSM 45590=KCTC 19843).

## Description of *Mycobacterium vinylchloridicum* (Cortés-Albayay *et al.* 2023) comb. nov.

vi.nyl.chlo.ri’di.cum. N.L. neut. adj. *vinylchloridicum*, related to vinyl chloride.

Basonym: *Mycolicibacterium vinylchloridicum* Cortés-Albayay *et al.* 2023 [88,95].

The description is as given for the basonym [88]. The type strain is L1 (=CECT 8761=DSM 6695).

## Description of *Mycobacterium xanthum* (Pan *et al.* 2022) comb. nov.

xan’thum. Gr. masc. adj. *xanthos*, yellow; N.L. neut. adj. *xanthum*, yellow.

Basonym: *Mycolicibacterium xanthum* Pan *et al.* 2022 [83,92].

The description is as given for the basonym [92]. The type strain is Y57 (=KCTC 49711=MCCC 1K04875).

## The future of LoRN

LoRN version 1.0.0 includes all names validly published under the ICNP [6] with a risk group classification [10] >1 in, at least, one country for some species in the same genus, or names of taxa classified by LPSN [5] as, at least, potentially pathogenic based on previous work [111] and LPSN’s own work to keep this list up to date based on the effective publications. This is the most comprehensive selection considered so far by CoMicProN [19]. Potential additions include prokaryotes of phytopathological or industrial relevance; the adaptation of such additions will depend on user interest and available resources. Regular updates of LoRN will be carried out by LPSN [5] in collaboration with CoMiCProN [19].

CoMiCProN considers the workflow [22] to be a fairly mature product. However, its application requires an assessment of criteria such as monophyly, salience and known practical implications, which may go wrong in individual situations. Furthermore, the details of the application of these criteria were deliberately not fixed in the workflow to allow for adaptation in response to user feedback. In addition to providing such feedback, we would also like to ask users to bear in mind that names in taxonomic suspension will eventually be changed and to prepare accordingly. We would also be grateful if users could inform us of any obstacles to the use of names from LoRN.

The same applies to databases and scientific journals. The benefits of LoRN would be greatest the more widespread the use of the recommended names. Editors and reviewers of journals, particularly those dealing with human or veterinary medical microbiology, are encouraged to follow the CoMiCProN guidelines [19] and to follow LoRN [22] when deciding which name to apply to a particular prokaryote. Curators of databases are therefore strongly encouraged to use LoRN [22]. Problems in doing so may well be temporary, and CoMiCProN would be happy to assist.

## Supplementary material

10.1099/ijsem.0.006943 Uncited Supplementary Material 1.

## Abbreviations

CoMiCProN: Ad Hoc Committee on Mitigating Changes in Prokaryotic Nomenclature
ICNP: International Code of Nomenclature of Prokaryotes
ICSP: International Committee on Systematics of Prokaryotes
LoRN: List of Recommended Names
LPSN: List of Prokaryotic names with Standing in Nomenclature
