# Supplementary material for: List of Recommended Names for bacteria of medical importance: report of the Ad Hoc Committee on Mitigating Changes in Prokaryotic Nomenclature
Source: Int J Syst Evol Microbiol. 2025 Oct 23;75(10):006943. doi: 10.1099/ijsem.0.006943 (PMC12548757; doi:10.1099/ijsem.0.006943)
Supplement: Uncited Supplementary Material 1. [file ijsem-75-06943-s001.pdf]

Mitigating changes in prokaryotic nomenclature: Definitions and Workflow

Introduction

The purpose of this document is to compile terms and their definitions for the further work of the *Ad Hoc* Committee on Mitigating Changes in Prokaryotic Nomenclature (CoMiCProN) and to describe the entire workflow for dealing with taxonomic proposals, making use of the defined terms.

Accurate language is of great importance in science and related fields; sloppy wording is usually the result of sloppy thinking. For this reason, these principles on which the work of CoMiCProN is based are presented as a set of definitions of terms, emphasising the interconnectedness of all the concepts involved. Cross reference are indicated with an arrow (→). The workflow is presented afterwards.

The purpose of this document is not to explain in detail the terms already used in prokaryotic nomenclature, particularly those defined in the → ICNP. Explanatory material has been provided previously. Official publications on prokaryotic nomenclature have been published continuously for decades. Both the List of Prokaryotic names with Standing in Nomenclature (LPSN) and the → ICSP website provide a list of these publications.

Definitions

Antitaxonomic views

Antitaxonomic thinking involves a combination of several of the following views. First, a firm belief in the → taxonomic *argumentum ad novitatem*, disapprovingly. This is linked to the belief that the rejection of names by the → Judicial Commission is an appropriate means of resolving the perceived problems with "name changes". Second, the view that taxonomists (rather than authors of subsequent publications, databases or other agencies) are responsible for "name changes", and that "name changes" are primarily about naming, not grouping. This is linked to the idea that taxonomic work should be subject to → pre-publication censorship. Third, the view that even if → monophyly is the main overall taxonomic criterion, a classification that overrides this criterion may be taxonomically superior in certain groups of organisms, regardless of consistency.

Further reading: this document; CoMiCProN guidelines.

Correct name

The name to be applied to a taxon under the given circumstances as defined by the → ICNP. This depends, among other things, on the → valid publication and → legitimacy of a name. Beware of the → taxonomic *argumentum ad novitatem*. The counterparts, if any, of the correct name are not “incorrect names” but synonyms.

Further reading: ICNP (Oren et al., 2023) and other official publications on nomenclature (listed in Arahal et al., 2023); CoMiCProN guidelines.

Note: “correct” should be treated as a reserved word; “incorrect” should not be used.

Hypertaxonomic views

Hypertaxonomic thinking involves a combination of several of the following views. First, a firm belief in the → taxonomic *argumentum ad novitatem,* approvingly. Second, the belief that it is beneficial to introduce taxonomic criteria in addition to → monophyly, such as → signaturism or → thresholdism, regardless of whether this leads to more → taxonomic proposals for reclassification. Third, the stance that it must be made easier for taxonomists to → validly publish names. (This may even involve proposing a competing code of nomenclature if the → ICNP remains adamant that certain names are not → validly published.) Other criteria, such as the concerns of those who have to use taxon names, or an interest in maintaining scientific standards or ensuring scientific reproducibility, are less important.

Hypertaxonomic thinking is typically the perspective of certain taxonomists. Weaker versions of the hypertaxonomic stance are held by the curators of some databases. In particular, the → taxonomic *argumentum ad novitatem* is attractive to databases because it is easy to implement.

Further reading: this document; CoMiCProN guidelines.

ICNP

The International Code of Prokaryotic Nomenclature, published by the→ ICSP. Defines, among many other things, the criteria for the → valid publication of a taxon name and for being the → correct name of a taxon. Document on which decisions of the → Judicial Commission are based. Not responsible for “name changes” other than the replacement of not validly published names with → validly published names or illegitimate names with → legitimate names.

Further reading: ICNP (Oren et al., 2023) and other official publications on nomenclature (listed in Arahal et al., 2023 and on LPSN).

ICSP

The International Committee on Systematics of Prokaryotes is a committee of the Bacteriology and Applied Microbiology Division of the International Union of Microbiological Societies (IUMS), composed of appointed representatives from national microbiological societies and co-opted members. Publishes the → ICNP.

Further reading: ICSP website, ICSP FAQ and ICSP statutes (Whitman et al., 2019).

Judicial Commission

A subsidiary of the → ICSP which issues Judicial Opinions in response to a Request for an Opinion. Bound in its decisions by the wording of the → ICNP. A Request for an Opinion may be approved or denied. Judicial Opinions may conserve or reject names or epithets, which affects which names can be considered → correct names. The criteria for rejecting a name or epithet are defined by the → ICNP. It is easier to conserve a name or epithet. However, such measures are only effective under certain conditions.

Further reading: ICNP (Oren et al., 2023) and other official publications on nomenclature (listed in Arahal et al., 2023 and on LPSN).

Note: “conserve”, “reject “, “ approve” and “deny” refer to actions of the Judicial Commission and should be treated as reserved words.

Known practical implications (of a taxon name)

A taxon name has known practical implications if the inferred presence of a taxon with that name requires practitioners to take action. These actions may be very different from those taken when a taxon with a different name is inferred to be present instead. If this is the case, the two names have considerably different known practical implications. For example, different bacterial names resulting from a clinical diagnosis may lead to different treatments for the patient.

The known practical implications of a name should not be confused with its → salience. While two names with considerable salience may have the same or different known practical implications, a name with no salience has no known practical implications.

Note: “significant” is often used to describe the results of statistical tests, so we use “considerable” here.

Legitimate name

Taxon name → validly published under the → ICNP and not in violation of any ICNP rules. Being legitimate is a necessary (but not sufficient) condition for being the → correct name. Validly published names that violate at least one ICNP rule are called illegitimate. For example, a later homonym of a validly published name is illegitimate.

Further reading: ICNP (Oren et al., 2023) and other official publications on nomenclature (listed in Arahal et al., 2023 and on LPSN).

Note: “legitimate” and “illegitimate” should be treated as reserved words.

Monophyly

A taxon is monophyletic according to a rooted phylogenetic tree if it includes all descendants of the common ancestor of the organisms included in the taxon. This is the main criterion for converting a rooted phylogenetic tree into a taxonomy: all taxa must be monophyletic according to that phylogenetic tree. Not enforced by the → ICNP but a widely accepted taxonomic criterion for decades. The monophyly criterion is relatively tolerant, as there are many ways of splitting a rooted phylogenetic tree into monophyletic taxa.

Further reading: Hennig (1950, 1965, 1966); Hull (1964); Wiley et al. (1991); Wiley and Lieberman (2011); Klenk and Göker (2011); Göker (2021); De Hoog et al. (2023).

New combination

Mainly at the species level. In the → ICNP (and all other nomenclature codes), a species name is the combination of the name of the genus to which the species belongs and a species-specific epithet. Reclassifications at the genus level usually result in such a → taxonomic proposal. When splitting or merging genera is proposed, it must be proposed that some species be placed in a different genus. This in turn results in the need to propose a new combination. A new combination may become → validly published, but this does not mean that it has to be considered the → correct name.

Further reading: ICNP (Oren et al., 2023) and other official publications on nomenclature (listed in Arahal et al., 2023 and on LPSN).

Parataxonomic views

Parataxonomic views are an alternative to the excesses of → antitaxonomic and → hypertaxonomic views. Parataxonomic views are fully compatible with the → ICNP. They do not aim at → pre-publication censorship, but they do advocate → taxonomic conservatism. When considering the adoption of a newer name that is → validly published and → legitimate as the → correct name, a parataxonomic committee would first determine whether the newer classification should be considered the better classification. For example, if the newer classification conservatively creates → monophyletic taxa from non-monophyletic ones, then the newer classification is taxonomically preferable.

If so, a parataxonomic committee would then determine whether there are objective reasons to apply → taxonomic suspension and to defer application of the newer classification. Objective reasons are based on the → salience of a name and go beyond the mere unwillingness of certain practitioners to adopt a particular classification. For example, an older name may be associated with legal restrictions, while its proposed replacement has not yet been evaluated by the appropriate regulatory agency. By keeping track of taxonomic proposals on the one hand and official regulations on the other, a parataxonomic committee would make recommendations as to which name should be adopted by practitioners for a given taxon at a given time.

Further reading: this document; CoMiCProN meeting minutes.

Note: this entry, of course, describes the work of CoMiCProN.

Pre-publication censorship

The idea that, beyond normal peer review, scientific freedom should be suppressed when it comes to taxonomic work. Usually based on the → taxonomic *argumentum ad novitatem* combined with a fear of “name changes”. Fails to take proper account of the fact that taxonomic proposals are a relatively innocent kind of activity, which in themselves do not lead to “name changes”. Typical symptom of → antitaxonomic views.

Further reading: this document.

Salience (of a taxon name)

The salience of a taxon name is a function of how well it is represented in official documents that have practical implications. For example, if an older name is associated with legal restrictions, and its proposed replacement has not yet been evaluated by the appropriate regulatory agency, then the older name has considerably greater salience. This may call for → taxonomic suspension.

The salience of a name should not be confused with its → known practical implications. While a name without salience also lacks known practical implications, two names with considerable salience may have the same or different known practical implications.

Note: “significantly” is often used for describing the results of statistical tests, hence we here use “considerably”.

Signaturism

The idea that, in addition to being → monophyletic, taxa must have a particular character signature, such as a particular set of "conserved signature indels". Applying the → monophyly criterion alone is more tolerant. Thus, when applied at the genus level, signaturism leads on average to more proposals for → new combinations than → taxonomic conservatism. This becomes particularly difficult to justify when a genus that already had a signature is split into smaller genera just because each of them also has a signature.

Further reading: this document; Göker (2021).

Taxonomic *argumentum ad novitatem*

An *argumentum ad novitatem* (appeal to novelty) is the logical fallacy that newer means better. The taxonomic *argumentum ad novitatem* is the belief that the last → validly published and → legitimate name (among a set of homotypic synonyms) must be treated as the → correct name according to official nomenclature. But those official rules, the → ICNP, say nothing of the sort.

Further reading: this document; CoMiCProN guidelines.

Taxonomic conservatism

Approach that attempts to repair taxa in a way that results in the least number of reclassifications. Taxonomic conservatism is part of the decision making of a → parataxonomic committee. It should not be confused with → taxonomic suspension. A parataxonomic committee would decline all taxonomic proposals that are not taxonomically conservative.

When applied at the genus level, taxonomic conservatism is limited to repairing taxa that are not → monophyletic. It does so by reassigning species to genera in a way that results in the lowest number of → new combinations. Therefore, taxonomic conservatism leads on average to fewer → new combinations than → signaturism or → thresholdism. When applied at the species level, taxonomic conservatism repairs taxa that are not → monophyletic and also applies → thresholdism with a boundary equivalent to 70% DDH.

Further reading: this document; Göker (2021); De Hoog et al. (2023); CoMiCProN guidelines.

Taxonomic proposal

For the purpose of the work of a → parataxonomic committee, the taxonomic proposals considered are only those that result in at least one name that is → validly published and → legitimate according to the → ICNP. A taxonomic proposal may result in one to many→ new combinations.

Note: "propose" and "proposal" should be treated as reserved words, only to be used for taxonomic proposals.

Taxonomic suspension

Taxonomic suspension means postponing the application of a newer classification, even if that classification is considered to be significantly better than the current classification from a purely taxonomic point of view. Taxonomic suspension should be based on objective non-taxonomic criteria and may be part of the recommendations of a → parataxonomic committee. It should not be confused with → taxonomic conservatism.

Further reading: this document; CoMiCProN guidelines.

Thresholdism

The idea that, in addition to being → monophyletic, taxa must have a certain degree of divergence, such as a maximum amount of genomic dissimilarity or a maximum amount of phylogenetic depth. Applying the → monophyly criterion alone is more tolerant. Thus, when applied at the genus level, thresholdism leads on average to more proposals for → new combinations than → taxonomic conservatism. This is particularly difficult to justify while thresholds remain arbitrary.

At the species level, thresholdism, if applied carefully, may be the most conservative solution, as criteria such as DDH, dDDH or ANI and derivatives have been used consistently for decades. They are identical to, or attempt to be equivalent to, 70% DDH. Departing from these criteria may therefore lead to more proposals for name changes than retaining them. This would be the case even if these criteria were considered arbitrary. At the subspecies level, thresholdism is not as well established as at the species level, but its use is less likely to lead to proposals for name changes.

Further reading: this document; Göker (2021).

Validly published name

The criteria for the → valid publication of a taxon name are defined by the → ICNP. Being validly published is a necessary (but not sufficient) condition for being → legitimate, and being legitimate is a necessary (but not sufficient) condition for being the → correct name.

Further reading: ICNP (Oren et al., 2023) and other official publications on nomenclature (listed in Arahal et al., 2023 and on LPSN).

Identification key representing the workflow

Comments on the identification key

The identification key is, of course, not supposed to be manually applied in the future by any member of the committee, let alone by a medical or other practitioner. The workflow is supposed to be implemented via database support, with minimal manual interaction.

The numbering in the identification key below reflects the steps required to reach a conclusion. The path from one step to the next is indicated by an arrow (→). The decisions to be made are indicated by **a** and **b** respectively.

Dichotomous identification key

1a. The taxonomic proposal involves the splitting or merging taxa above genus rank or none of the taxon names involved meets the committee's selection criteria anyway. **Solution:** Beyond the scope of the committee.

1b. The taxonomic proposal involves the splitting or merging of subspecies, species or genera (or moving species from one genus to another without merging genera) and at least one of the taxon names affected meets the committee's selection criteria. → **2**

2a. The taxonomic proposal involves the splitting or merging of subspecies. **Solution:** The rest of the workflow is as for splitting or merging of species (→ **3**) after replacing “species” with “subspecies” and discarding the last two sentences of 8a.

2b. The taxonomic proposal involves the splitting or merging of species. → **3**

2c. The taxonomic proposal involves the splitting or merging of genera (or moving species from one genus to another without merging genera). → **10**

3a. The taxonomic proposal involves the splitting of a species. → **4**

3b. The taxonomic proposal involves the merging of species. → **6**

4a. The proposal to split a species is based on taxonomic conservatism. → **5**

4b. The proposal to split a species is not based on taxonomic conservatism. **Solution:** Permanently decline the proposal; treat newly proposed species names as later heterotypic synonyms of the older species name.

5a. The newly proposed species names do not differ considerably in salience from the older species name or the newly proposed species names differ considerably in their known practical implications. **Solution:** Accept the proposal. Announce the newly proposed names through the established channels, along with their known practical implications.

5b. The newly proposed species names differ considerably in salience from the older species name but the newly proposed species do not differ considerably in their known practical implications. **Solution:** Suspend application of the proposal. Announce the newly proposed names through the established channels. Adopt the proposal when the names are no longer considerably different in salience.

6a. The proposal to merge species is based on taxonomic conservatism. → **7**

6b. The proposal to merge species is not based on taxonomic conservatism. **Solution:** Permanently decline the proposal; continue to treat the species names involved as names of separate species.

7a. The species names proposed as heterotypic synonyms do not differ considerably in salience and do not differ considerably in their known practical implications. **Solution:** Accept the proposal. Announce the newly proposed synonymy through the established channels.

7b. The species names proposed as heterotypic synonyms differ considerably in salience or differ considerably in their known practical implications. → **8**

8a. The species names proposed as heterotypic synonyms differ considerably in their known practical implications. **Solution:** Suspend application of the proposal. Request the Judicial Commission to conserve the epithets of the species names involved against each other. Announce the situation through the established channels. If the Judicial Commission approves the Request, the proposal to merge the species is permanently declined. If the Judicial Commission denies the Request, propose subspecies names (new combinations), if not already proposed, to reflect the differences in known practical implications instead of the species names proposed as later heterotypic synonyms. Adopt the proposal to merge the species once the subspecies names have gained salience.

8b. The species names proposed as heterotypic synonyms differ considerably in salience but not in their known practical implications. → **9**

9a. One of the species names with the greatest salience has priority over the other species names involved. **Solution:** Accept the proposal. Announce the newly proposed synonymy through the established channels.

9b. The salience of the species name that has priority over the other species names involved is considerably lower than the highest salience of the species names involved. **Solution:** Suspend application of the proposal. Request the Judicial Commission to conserve the epithet of the species name with the highest salience involved over the other species names involved. Announce the situation through the established channels. If the Judicial Commission approves the Request, stop suspending application of the proposal and emphasize that the resulting correct name is now different. If the Judicial Commission denies the Request, adopt the proposal to merge the species once the salience of the species names involved is no longer considerably different.

10a. The taxonomic proposal involves splitting a genus. → **11**

10b. The taxonomic proposal involves the merging of genera (or moving species from one genus to another without merging genera). → **13**

11a. The proposal to split a genus is based on taxonomic conservatism. → **12**

11b. The proposal to split a genus is not based on taxonomic conservatism. **Solution:** Permanently decline the proposal; treat newly proposed genus names, if any, as later heterotypic synonyms of the older genus name; treat newly proposed species names (new combinations) as homotypic synonyms of the older species names (basonyms or older new combinations).

12a. The new species names (new combinations) proposed as a result of the transfer of species to another genus do not differ considerably in salience from the older species names (basonyms or older new combinations). **Solution:** Accept the proposal. Announce the newly proposed names through the established channels.

12b. The new species names (new combinations) proposed as a result of the transfer of the species to another genus differ considerably in salience from the older species names (basonyms or older new combinations). **Solution:** Suspend application of the proposal. Announce the newly proposed names through the established channels. Adopt the proposal when the names are no longer considerably different in salience

13a. The proposal to merge genera (or to move species from one genus to another without merging genera) is based on taxonomic conservatism. → **14**

13b. The proposal to merge genera (or to move species from one genus to another without merging genera) is not based on taxonomic conservatism. **Solution:** Permanently decline the proposal; treat genus names involved as heterotypic synonyms; treat newly proposed species names (new combinations) as homotypic synonyms of the older species names (basonyms or older new combinations).

14a. The new species names (new combinations) proposed as a result of the transfer of the species to another genus do not differ considerably in salience from the older species names (basonyms or

older new combinations). **Solution:** Accept the proposal. Announce the newly proposed names through the established channels.

14b. The new species names (new combinations) proposed as a result of the transfer of the species to another genus differ considerably in salience from the older species names (basonyms or older new combinations). **Solution:** Suspend application of the proposal. Announce the newly proposed names through the established channels. Adopt the proposal when the names are no longer considerably different in salience.

References

Arahal DR, Bull CT, Busse HJ, Christensen H, Chuvochina M, Dedysh SN, Fournier PE, Konstantinidis KT, Parker CT, Rossello-Mora R, et al., Guidelines for interpreting the International Code of Nomenclature of Prokaryotes and for preparing a Request for an Opinion. *Int J Syst Evol Microbiol* 2023; **73**:5782.

De Hoog S, Walsh TJ, Ahmed SA, Alastruey-Izquierdo A, Alexander BD, Arendrup MC, Babady E, Bai F, Balada-Llasat J, Borman A, Chowdhary A, Clark A, Colgrove RC, Cornely OA, Dingle TC, Dufresne PJ, Fuller J, Gangneux J, Gibas C, Glasgow H, Gräser Y, Guillot J, Groll AH, Haase G, Hanson K, Harrington A, Hawksworth DL, Hayden RT, Hoenigl M, Hubka V, Johnson K, Kus JV, Li R, Meis JF, Lackner M, Lanternier F, Leal Jr. SM, Lee F, Lockhart SR, Luethy P, Martin I, Kwon-Chung KJ, Meyer W, Nguyen MH, Ostrosky-Zeichner L, Palavecino E, Pancholi P, Pappas PG, Procop GW, Redhead SA, Rhoads DD, Riedel S, Stevens B, Sullivan KO, Vergidis P, Roilides E, Seyedmousavi A, Tao L, Vicente VA, Vitale RG, Wang Q, Wengenack NL, Westblade L, Wiederhold N, White L, Wojewoda CM, Zhang SX. A conceptual framework for nomenclatural stability and validity of medically important fungi: a proposed global consensus guideline for fungal name changes supported by ABP, ASM, CLSI, ECMM, ESCMID-EFISG, EUCAST-AFST, FDLC, IDSA, ISHAM, MMSA, and MSGERC. *J Clin Microbiol* 2023;**61**:e00873-23.

Göker M. What can genome analysis offer for bacteria? In: Bridge P, Smith D, Stackebrandt E (eds), Trends in the systematics of bacteria and fungi, CAB International, Wallingford, 2021, pp. 255-281.

Hennig W. Grundzüge einer Theorie der Phylogenetischen Systematik. Deutscher Zentralverlag, Berlin, 1950.

Hennig W. Phylogenetic systematics. *Annual Review of Entomology* 1965; **10**:97-116.

Hennig W. Phylogenetic systematics. University of Illinois Press, Urbana, 1966.

Hull DL. Consistency and monophyly. *Systematic Zoology* 1964; **13**:1-11.

Klenk HP, Göker M. En route to a genome-based classification of Archaea and Bacteria? *Systematic and Applied Microbiology* 2010; **33**:175-182.

Oren A, Arahal DR, Göker M, Moore ERB, Rossello-Mora R, Sutcliffe IC. International Code of Nomenclature of Prokaryotes. Prokaryotic Code (2022 Revision). *Int J Syst Evol Microbiol* 2023; **73**:5585.

Whitman WB, Bull CT, Busse HJ, Fournier PE, Oren A, Ventura S. Request for revision of the Statutes of the International Committee on Systematics of Prokaryotes. *Int J Syst Evol Microbiol* 2019; **69**:584-593.

Wiley EO, Lieberman BS. Phylogenetics. Theory and practice of phylogenetic systematics. Wiley-Blackwell, Hoboken (NJ), 2011, 406 pp.

Wiley EO, Siegel-Causey D, Brooks DR, Funk VA. The Compleat Cladist: A primer of phylogenetic procedures. University of Kansas Museum of Natural History, Lawrence (Kansas), 1991, 158 pp.

Imprint

Date of this document

2025-05-02
